# Cancer in Moroccan elderly: the first multicenter transverse study exploring the sociodemographic characteristics, clinical profile and quality of life of elderly Moroccan cancer patients

**DOI:** 10.1186/s12885-020-07458-0

**Published:** 2020-10-12

**Authors:** Mounia Amzerin, Mohamed Layachi, Aziz Bazine, Rachid Aassab, Samia Arifi, Zineb Benbrahim, Mohamed Reda Khmamouche, Mouna Kairouani, Hanan Raiss, Noura Majid, Saloua Ouaouch, Mohammed Ichou, Said Afqir, Nawfal Mellas, Mohamed Fetohi, Rachid Razine, Hassan Errihani

**Affiliations:** 1 Ahmad Bin Zayed Al Nahyan Center of Cancer Treatment, Department of Medical Oncology, Tangier, Morocco; 2 Mohamed V University in Rabat, Faculty of Medicine and Pharmacy of Rabat, Rabat, Morocco; 3 Mohamed VI University Hospital, Department of Medical Oncology, Marrakech, Morocco; 4 Moulay Ismail Military hospital, Department of Medical Oncology, Meknes, Morocco; 5 Hassan II Hospital, Department of Medical Oncology, Agadir, Morocco; 6 Hassan II University Hospital, Department of Medical Oncology, Fez, Morocco; 7 Mohamed V Military teaching Hospital, Department of Medical Oncology, Rabat, Morocco; 8 Hassan II Oncology Center, Department of Medical Oncology, Mohammed VI University Hospital, Oujda, Morocco; 9 Reginal Center of Oncology, Department of Medical Oncology, Al-Hoceima, Morocco; 10 Ibn Rochd University Hospital, Oncology Center, Department of Medical Oncology, Casablanca, Morocco; 11 Proximity Center of Oncology, Beni Mellal, Morocco; 12 Laboratory of Biostatistics, Epidemiology and Clinical Research, Mohammed V University in Rabat, Faculty of Medicine and Pharmacy of Rabat, Rabat, Morocco; 13 Department of Public Health, University of Mohamed V Rabat, Faculty of Medicine and Pharmacy of Rabat, Rabat, Morocco; 14 National Institute Of Oncology, Department of Medical Oncology, Mohamed V University in Rabat, Rabat, Morocco

**Keywords:** Geriatric oncology, Elderly, Cancer, Multicenter study, Morocco, G8, Religious practice

## Abstract

**Background:**

Moroccan incidence of cancer is increasing with the lengthening of life expectancy. Data regarding elderly Moroccan cancer patients are lacking. In the context of our project aiming to develop an adapted version of the Comprehensive Geriatric Assessment CGA to the Moroccan population, we launched the first Moroccan multicenter transverse study to explore the characteristics of elderly Moroccan cancer patients.

**Methods:**

The study was conducted in nine Moroccan medical oncology departments. Patients were enrolled over 4 months. Inclusion criteria were patients aged 65 years or over with verified solid cancer. The questionnaire included four sections: socio-demographic and economic data, clinical data, vulnerability and EORTC-QLQ C30. We explored the entire included population. Then, we compared the results according to age (65–70 years old and ≥ 71 years old) and sex. We also explored the correlation between G8 scores and the ability to practice religion as an indicator of fitness level.

**Results:**

In total, 164 patients were enrolled. The mean age was 73.18 ± 6.01 years. The majority of patients were married, lived with their children and received their financial income from them. Fifteen percent of families asked to hide the diagnosis from the patient. Breast (23%), colorectal (15.9%) and lung (14%) cancers were the most frequent, and 83.5% had an abnormal G8. The majority of the patients were independent for basic daily activities. Female patients had poorer social and economic conditions. Abnormal G8 was correlated with religious practice and quality of life scores.

**Conclusion:**

This is the first multicenter prospective study designed to collect data on the lifestyle and clinical profiles of elderly Moroccan cancer patients as an Arab and Muslim population. Our study shows that it is a well-cared-for population with strong social ties. However, there is deep economic vulnerability, especially among women, requiring urgent care. Religious practice is an important daily activity for our elderly patients and should be included in the Moroccan CGA.

## Background

Population aging is a growing issue worldwide. By 2050, 21% of the world population will be aged 60 or older, of whom more than three quarters will live in developing countries [1].

Morocco is a lower-middle income Muslim country located in the Maghreb Region of North Africa. It has a population of over 34,500,000, of whom 9.6% are aged 60 years old or over. The age pyramid is in transition due the lengthening of life expectancy and fertility decline, which are the result of medical system improvement, social change and easy access to contraceptives [2]. Most Moroccan elderly live in urban areas (59%), and 5.9% live alone [2].

In Morocco, there are two regional cancer registries: Casablanca and Rabat. According to the latest data, standardized incidence of cancer is 137.3 per 100,000 and 124.8 per 100,000 in the two regions, respectively [3, 4]. Breast cancer (20%), lung cancer (11.4%) and colorectal cancer (6.7%) are the most frequent [3, 4]. Lung cancer and prostate cancer are the most common among men, and breast cancer and cervical cancer are the most frequent in women. Verified data regarding cancer mortality are missing [3, 4].

In Morocco, it is expected that the elderly will represent 23.2% of the population by 2050 versus 9.4% in 2014 [5] . The aging of the Moroccan population urges the government to anticipate and develop adapted strategies. There is an important lack of management of the elderly. There are 3 residencies dedicated entirely to the elderly in the whole country managed by nonprofit associations [6]. There is no national diploma for specialization in geriatrics in Morocco. Geriatric care is mostly practiced by internists and a few private geriatricians who did their specialization abroad. The Faculty of Medicine of Rabat has launched a one-year certificate training since 2014–2015.

Since the new reform of health insurance was established in 2005, there have been two principal medical coverage regimens for the Moroccan population: AMO (Assurance Maladie Obligatoire, Mandatory Health Insurance) and RAMED (Régime d’Assistance MEDicale, Medical Assistance Scheme). AMO covers public sector and private sector employees through CNOPS (Caisse Nationale des Organismes de Prévoyance Sociale, a national fund for social welfare organizations) and CNSS (Caisse Nationale de Sécurité Sociale, a national social security fund regimen). RAMED covers poor people with an income of less than MAD 300 (US$34) per capita per month [7]. Moroccan seniors benefit from health coverage according to their social status. Cancer patients who are in need, in addition to the healthcare provided by RAMED, benefit from the ACCES program, launched by the Lalla Salma Foundation, which allows free access to cancer treatment [8].

It is well established that cancer is a disease of aging [9, 10]. Cancer perception by older patients and treatment decisions are influenced by cultural issues [11]. Therefore, management of older patients with cancer should also take in consideration their cultural background. A comprehensive geriatric assessment (CGA) is strongly recommended by International Society of Geriatric Oncology for older patients [12]. We launched a project aiming to create an adapted Moroccan version of the CGA. We started by collecting objective information through the first Moroccan transverse study to describe the socio-demographics, clinical characteristics and quality of life of our older cancer patients. In this paper, we report the first results of this project.

There is a large Moroccan Diaspora all over the world, especially in Europe, which is the principal destination for Moroccan emigrants. Moroccans are known for retaining their original lifestyle to not ‘lose’ their identity [13]. Thus, the management of elderly Moroccan patients with cancer would be easier for international physicians if they had more data about their patients’ culture and lifestyle. Moreover, elderly Moroccans have many characteristics in common with other Arab populations, especially in religion and social habits. This kind of study could help to better understand and manage all older Arab patients with cancer.

## Methods

### Design

**Fig. 1:**
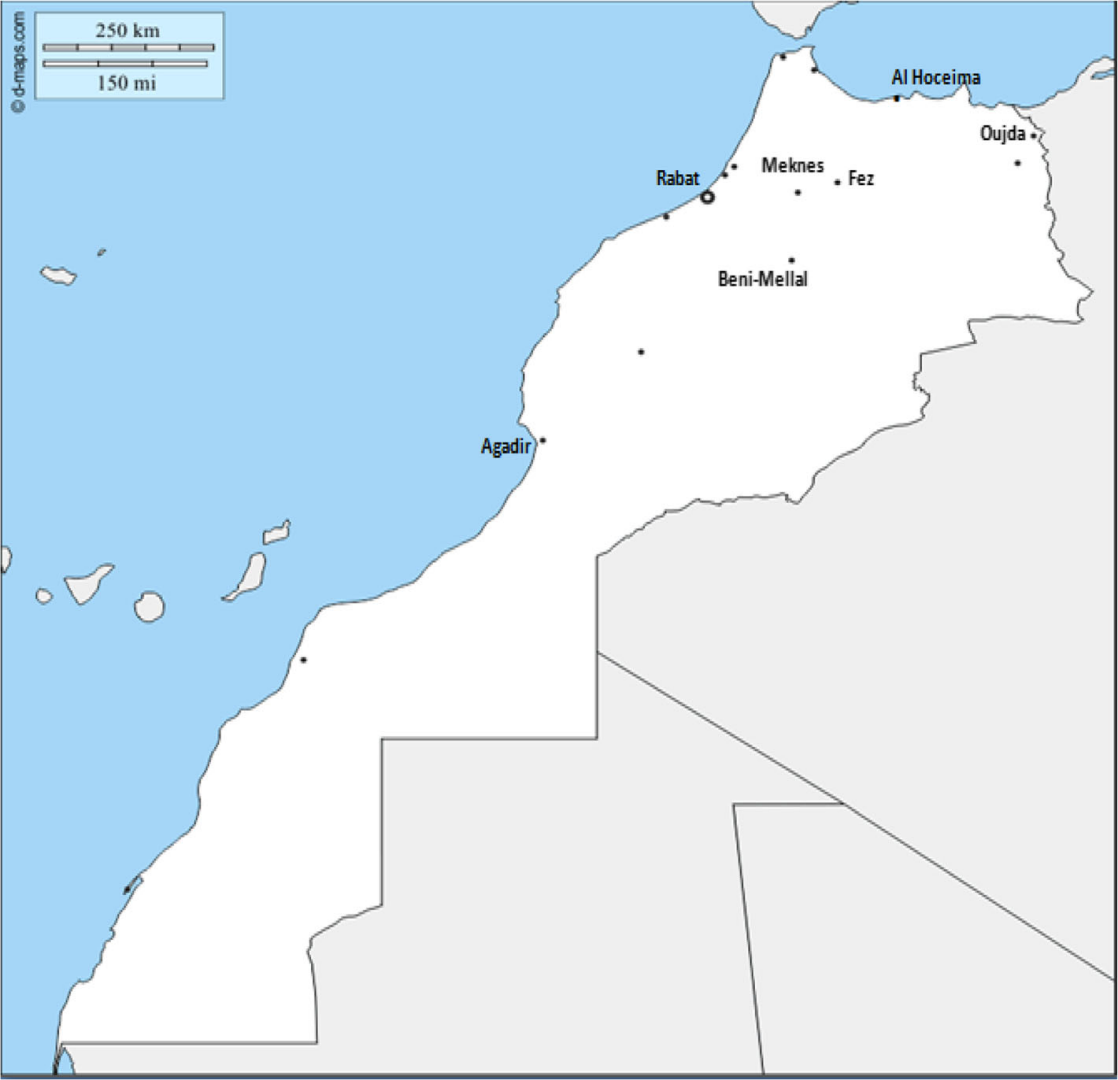
Morocco map showing the location of the departments of Medical Oncology included in the study. The included centers are located in the following cities: Al Hoceima, Oujda, Fez, Meknes, Rabat, Beni-Mellal and Agadir. The base map has been taken, with permission, from the website https://d-maps.com/carte.php?num_car=22750&lang=fr

We conducted a cross-sectional multicenter study in 9 Moroccan public, civil and military medical oncology departments (Fig. 1). The enrollment lasted 4 months from June 15th to October 15th, 2015. A specific questionnaire was developed for this study (An English language version is available in the supplementary material).

### Patients

Inclusion criteria: The study aimed to include all patients aged 65 years or older diagnosed with solid cancer. Every eligible patient who presented to the department for a scheduled consultation, chemotherapy session or emergency was asked to be included in the study. Hospitalized patients were also eligible.

The study was conducted only in medical oncology departments. Thus, it will not be representative of Moroccan epidemiology. Patients with localized head and neck, lung and cervical cancers are treated in radiotherapy departments, so they were not included.

All included patients signed an informed consent.

Exclusion criteria: Patients with a performance status of 4.

### Questionnaire

We developed a questionnaire of 4 sections: socio-demographic and economic data, clinical data, vulnerability and quality of life assessment. Clinical data were filled in by investigators from patient medical records. Other sections were filled in by the patients. For illiterate patients, investigators asked them the questions and filled their responses in the questionnaire. The questionnaire was administered once for each patient included, and it took between 15 and 30 min. The treatment plan was not reviewed after the patient was included in the study.

#### Socio-demographic and economic data

This section explored patient demographic and social information including age, sex, living conditions (urban, rural, alone, with spouse, with children, with brother/sister, in an institution), religion, civil status, number of children, level of education, source of financial income (personal pension, spouse pension, aid from children, still working), monthly income and health care coverage.

The education level of the patients was divided into illiterate and who had mosque, elementary, high school or university education. Mosque education consists of learning Arabic, the Quran and religion sciences. It has represented the main method of education for Moroccans for centuries.

Regarding income, we referred to the classification made by the government, and we have adapted it to the income ranges reported by the patients to be more representative of the poor. According to High Commission for Planning, the middle class has an income between 2800 Dirhams (252 Euros) and 6760 dh per month (610 Euros) [5]. We used the following ranges:

- < 1500 dh per month (136 Euros).

− 1500–3000 dh per month (136–273 Euros).

− 3000–5000 dh per month (273–455 Euros).

− 5000–8000 dh per month (455–728 Euros).

- > 8000 dh per month (728 Euros).

In the Moroccan context, it is usual for the family to ask the doctor to hide the diagnosis of cancer from the patient [14], so we included this question in the questionnaire.

This section also included information about toxic habits: smoking, alcoholism and medicinal plant consumption.

#### Clinical data

To explore comorbidities, we looked for the most frequent ones. The age-adjusted Charlson Comorbidity Index aaCCI (Age-adjusted Charlton comorbidity Index) was also calculated [15]. We explored falls by asking patients and cancer data from medical reports.

#### Vulnerability

In the vulnerability section, we used the G8 screening tool [16].

The activities of daily living (ADL) were evaluated through the EORTC QLQ-C30 questionnaire by exploring the role, physical, cognitive and emotional function scores. In addition, we asked for each daily habit separately. We explored the degree of dependency for toileting, bathing, dressing, eating, and walking indoors and outdoors.

We included also religious practice as it represents an indispensable daily activity and can be good indicator of the vulnerability level of the patients. Ablutions and prayer are practiced five times a day at specific times. Ablution is mandatory before each prayer. Fit people do wet ablutions by washing their hands, forearms, face and feet one to three times each. If tired, they only use purified sand or dust to wipe their face and hands, which is considered dry ablution. Each prayer can take between 5 and 10 min according to the number of units. Every unit consists of reciting Quran verses of followed by movements including (in this order) bowing low with hands on knees, standing, prostration, sitting, prostration and standing up while reciting well-defined prayers. There are one to four units in each prayer. Unfit people can pray sitting or lying as normal prayer requires a certain level of physical condition; thus, they do not make these movements, and they only recite the Quran and prayers. Ramadan fasting is the third pillar of Islam. It consists of abstinence from food, drink and sexual activity from dawn to sunset during 1 month each year. Tired people or the elderly may not fast if their health will be compromised. In addition to Ramadan, very fit people can do optional fasting as much as they want, such as 1 day per month or two per week and/or during some religious holidays.

#### EORTC QLQ-C30

To assess the quality of life, we used the Moroccan validated version of the EORTC QLQ C30 questionnaire (Version 3.0) [17], which includes five functional scales (physical, role, cognitive, emotional, and social), three symptom scales (fatigue, pain, and nausea and vomiting), six single items (dyspnea, insomnia, appetite loss, constipation, diarrhea, and financial difficulties) and a global health and quality-of-life scale.

Score calculations were made according to the scoring recommendation [18].

### Statistical analysis

The statistical analysis was performed using SPSS 13.0. We used a Chi-square test to compare the qualitative variables and Student’s t-test or the Mann-Whitney U test to compare quantitative variables. The difference between subgroups was considered significant when the *p*-value was less than 0.05.

We explored the entire population included. Then, we made comparisons according to age (65–70 years old vs ≥71 years old), sex, G8 score, medical coverage and sociodemographic factors. The study was approved by the ethical committee of the University of Medicine and Pharmacy of Rabat.

## Results

In total, 165 patients were asked to be included in the study, and 164 patients were accepted and enrolled. Two patients were not included because they had a performance status at 4. In the Regional Cancer Center of Al Hoceima, all eligible patients were included. These patients represented 14% of the total included patients.. In addition to Al Hoceima, information regarding the proportion of elderly among all patients was available for Meknes (Table 1). The mean age was 73.18 ± 6.01 years old, and almost the two thirds were aged 70 or over. All of the patients were Muslim, and the majority were married (62.5%) and had at least 4 children (71.8%). Approximately half were illiterate, lived with their children, received their financial income from them and received less than 1500 dh (126 Euros) in monthly income. One third of patients had a mutual health insurance (public, military), and 61% were affiliated with RAMED. More than three quarters came accompanied to the hospital, and 15% of the families asked to hide the diagnosis from the patient. Regarding substance use, 2.5% were active smokers, no one were consuming alcohol at the time of the study, and the use of medicinal plants was reported by 12.2% of patients.

**Table 1:** Socio-demographic characteristics of the whole population included

	N	%
**Center**		
**-University Hospital**	63	38.4
**-Regional Center of Oncology**	63	38.4
**-COP**	3	1.8
**-Military Hospital**	35	21.3
**Representativity**		
**-Al Hoceima**	37	14%
**-Meknes**		
*Military	27	21%
**Context**		
**-Day hospital**	59	45.7
**-Consultation**	52	40.3
**-Hospitalization**	17	1.2
**-Emergency**	1	0.8
**Age**		
**- > 70 years old**	101	62
**Sex**		
**-Male**	84	51.2
**-Female**	80	48.8
**Living**		
**-Urban**	106	65
**-Rural**	57	35
**Religion**		
**-Muslim**	164	100
**Civil status**		
**-Single**	2	1.3
**-Married**	100	62.5
**-Divorced**	3	1.9
**-Widower**	55	34.4
**Number of children**		
**-0**	12	7.7
**-1–3**	32	20.5
**-4–6**	56	35.9
**- > 6**	56	35.9
**Level of education**		
**-Illiterate**	87	53.4
**-Mosque**	32	19.6
**-Elementary**	24	14.7
**-High school**	17	10.4
**-University**	2	1.2
**Personal pension**	39	24.2
**Spouse pension**	19	11.8
**Financial aid from children**	85	52.8
**Still working**	11	6.8
**Other**	34	21.1
**Monthly income in MAD (euros)**		
**- < 1500 (136)**	72	46.2
**-1500–3000 (136–273)**	42	26.9
**-3000–5000 (273–455)**	18	11.5
**-5000–8000 (455–728)**	11	7.1
**- > 8000 (728)**	13	8.3
**Medical coverage**		
**-Ramed**	100	61.3
**-CNOPS (Public sector)**	12	7.4
**-CNSS (Private sector)**	14	8.6
**-Military**	29	17.8
**-Private insurance**	3	1.8
**-None**	5	3.1
**Living alone**	7	4.3
**Living with spouse**	72	44.4
**Living with children**	100	61.7
**Living with brother/sister**	6	3.7
**Living in an institution**	0	0
**Accompanying to the hospital**		
**-None**	22	13.7
**-1 person**	98	60.9
**- > 1 person**	41	25.5
**Request by the family to hide the diagnosis from the patient**	25	15.3
**Smoking**		
**-Never**	94	57.7
**-Former**	52	31.9
**-Active**	4	2.5
**-Passive**	13	8
**Alcohol use**		
**-Ne er**	147	89.6
**-Former**	17	10.4
**-Active**	0	0
**Use of medicinal plants**	20	12.2

Breast (23%), colorectal (15.9%) and lung (14%) cancers were the most frequent localizations. Forty-four percent of the patients had metastatic disease from the outset, and the treatment was palliative in 65% of cases. Treatment was adapted to age/comorbidities for 19.2% of patients, and systematic use of G-CSF was seen in 3.1% of cases. Meanwhile, 2.5% of patients were treated with best supportive care exclusively (Table 2).

**Table 2:** Clinical data in the whole population included

	N	%
**Hypertension**	50	30.5
**Diabetes**	42	25.6
**Dyslipidemia**	11	6.7
**Cardiopathy**	9	5.5
**Osteoporosis**	24	14.7
**Arthrosis**	40	24.4
**Renal insufficiency**	12	7.3
**CCI ≥ 2**	23	14.2
**CCI ≥ 3**	11	6.8
**Fracture in the previous year**	8	4.9
**Fall in the previous year**	54	32.9
**Complication**		
**-Minor**	42	83.7
**-Major**	7	14.3
**Medications/day**		
**-0**	28	18.9
**- < 3**	81	54.7
**- ≥ 3**	39	26.4
**Performance status**		
**- < 2**	87	53.7
**- ≥ 2**	75	46.3
**Pain**		
**-Median**	3.21 [0;5]	
**-Pain free**	56	35.4
**-Mild**	47	29.7
**-Moderate**	40	25.3
**-Severe**	15	9.5
**Localization**		
**-Breast**	38	23.2
**-Colorectal**	26	15.9
**-Lung**	23	14
**-Stomach**	13	7.9
**-Prostate**	13	7.9
**-Ovary**	10	6.1
**-Bladder**	9	5.5
**-Other**	32	19.5
**Stage**		
**-Localized**	41	25.2
**-Locally advanced**	22	13.5
**-Metastatic**	72	44.2
**-Recurrence**	28	17.1
**Status**		
**-First consultation**	10	6.1
**-Workup**	13	8
**-Receiving treatment**	117	71.8
**-Follow up**	23	14.1
**Chemotherapy**	140	88.6
**Radiotherapy**	14	8.9
**RCC**	5	3.2
**Hormonal therapy**	10	6.3
**Best supportive care**	4	2.5
**Refusal of intraveinous treatment**	5	3
**Type of treatement**		
**-Standard**	130	80.7
**-Adapted**	31	19.2
**Systematic GCSF**	5	3.1
**Strategy**		
**-Curative**	57	34.8
**-Palliative**	107	65.2

The most frequent comorbidities were hypertension (30.5%), diabetes (25.6%) and arthrosis (24.4%). Only 6.8% of patients had an aaCCI ≥3. About one third of patients had at least one fall during the previous year with minor complications for 83% of them. Polymedication was reported by 26% of patients, 46% had a PS ≥ 2 and almost 30% were pain free.

The G8 score was abnormal in 83.4% of patients. The majority of patients were independent in their daily habits. Total dependency was seen in 8.5% for toileting, 9.8% for clothing, 16.5% for bathing, 7.4% for walking indoors and 7.4% for walking outside. Only 12.3% could make errands and cook for themselves, while 75.3% were totally dependent on their relatives for cooking. Less than 20% of patients were sexually active (Table 3).

**Table 3:** Frailty and daily habits in the whole population included

	N	%
**G8**		
**-Mean**	10.87 ± 3.23	
**- > 14**	26	16.6
**- ≤ 14**	131	83.5
**Toilet**		
**-Independent**	126	76.8
**-Partially dependent**	24	14.6
**-Totally dependent**	14	8.5
**Clothing**		
**-Independent**	113	68.9
**-Partially dependent**	35	21.3
**-Totally dependent**	16	9.8
**Bath**		
**-Independent**	84	51.2
**-Partially dependent**	53	32.3
**-Totally dependent**	27	16.5
**Walking indoor**		
**-Independent**	113	69.3
**-Partially dependent**	38	23.3
**-Totally dependent**	12	7.4
**Walking outdoor**		
**-Independent**	82	50
**-Partially dependent**	49	29.9
**-Totally dependent**	33	20.1
**Food**		
**-Cooking + errands**	20	12.3
**-Cooking**	20	12.3
**-Cooking made by another person**	122	75.3
**Number of meals per day**		
**-3 meals + snacks**	64	39
**-3 meals**	61	37.2
**-Less than 3 meals**	39	23.8
**Ablutions**	146	93.6
**Wet ablutions**	98	62.8
**Dry ablutions**	56	35.9
**Prayer**	142	86.6
**Prayer standing**	47	28.7
**Prayer sitting**	102	62.2
**Prayer Lying**	12	7.3
**Ramadan fasting**		
**-No**	116	71.2
**-Yes**	47	28.8
**Optional fasting**	35	21.3
**Sexual activity**	30	18.4

The large majority was practicing religion. Wet ablutions were performed in 62.8% of the study sample; meanwhile, 69.5% could not perform the standing prayer, so they prayed in a sitting or lying position. Seventy-one percent of patients could not fast during Ramadan for health issues while 21% did optional fasting.

Patients had medium scores for physical, role and cognitive functions. Emotional function was average with a median of 66.67 [33, 33;83.33] ranging from 0 to 100. Social function was not altered for the majority of patients, ranging from 16.67 to 100 with a median of 100 [66.67;100]. “Financial difficulties” was the most present symptom among patients with a median of 100 [33.33; 100]. Other important symptoms were pain, dyspnea, insomnia and anorexia (33.33 [00; 66.67]) (Table 4).

**Table 4:** QoL scores in the whole population included

	Median	Mean
**Functions**		
**-Physical function**	46.43 [20;73.33]	47.43 ± 32.36
**-Role function**	50 [00;66.67]	44.88 ± 37.23
**-Cognitive function**	66.67 [50;83.33]	68.20 ± 28.91
**-Emotional function**	66.67 [33,33;83.33]	61.86 ± 31.77
**-Social function**	100 [66.67;100]	79.75 ± 31.08
**Symptoms**		
**-Fatigue**		55.27 ± 33.99
**-Nausea. Vomiting**	0 [00;33.33]	18.81 ± 28.63
**-Pain**	33.33 [00;66.67]	41.10 ± 35.82
**-Dyspnea**	33.33 [00;66.67]	29.24 ± 34.50
**-Insomnia**	33.33 [00;66.67]	34.35 ± 37.48
**-Anorexia**	33.33 [00;66.67]	38.24 ± 39.41
**-Constipation**	0 [00;33.33]	23.72 ± 33.27
**-Diarrhea**	0 [00;33.33]	11.24 ± 23.48
**-Financial difficulties**	100 [33.33;100]	69.12 ± 39.29
**Global QoL Score**		52.20 ± 25.87

Older patients had significantly more arthrosis (31.7 vs 11.3% p 0.003) and falls in the previous year (38.6% vs 22.6% p 0.03). Their aaCCI was higher (3.93 ± 1.16 vs 2.64 ± 1.03 *p* < 0.001) while their predicted 1-year survival, calculated with aaCCI, was lower (77.03% ± 6.59% vs 85.27 ± 6.99% *p* < 0.001). They were significantly more dependent for cooking (83% vs 63.9% p 0.01). They did more sitting prayer (62.6% vs 50%, p: 0.001), but no difference was observed in fasting. There were no differences in G8 score, daily habits, ADL scores, emotional function and social function between the two subgroups. Older patients had higher scores of financial difficulties (Table 5).

**Table 5:** Significant difference in arthrosis, predited survival, falls, prayer and financial difficulties according to age

	65–70 N (%)	≥ 71 N (%)	P
**Arthrosis**	7 (11.3)	32 (31.7)	0.003
**Predicted one year survival**	85.27 ± 6 .99	77.03 ± 6.59	< 0.001
**Fall in previous year**	14 (22.6)	39 (38.6)	0.03
**Prayer sitting**	31 (50)	70 (62.6)	0.01
**Financial difficulties**	51.66 ± 42.54	71.36 ± 38.40	0.03

Significant differences were observed between men and women. There were more widows among women and more married people among men. Women had less education, personal pension, income and medical cover. No woman had active smoking or alcohol consumption in her medical history. Men had less osteoporosis, were more dependent for cooking and more sexually active (Table 6). Financial difficulties were significantly more pronounced among women. No difference was seen in the other functions.

**Table 6:** Significant differences in sociodemographic characterisctics, toxic habits, cooking and financial difficulties according to sex

	Male N (%)	Female N (%)	P
**Civil status**			< 0.001
**-Single**	1 (1.2)	1 (1.3)	
**-Married**	73 (89)	27 (34.6)	
**-Divorced**	1 (1.2)	2 (2.6)	
**-Widower**	7 (8.5)	48 (61.5)	
**Education**			< 0.001
**-Illiterate**	20 (24.1)	67 (83.8)	
**-Mosque**	27 (32.5)	5 (6.3)	
**-Elementary**	19 (22.9)	5 (6.3)	
**-High school**	15 (18.1)	2 (2.5)	
**-University**	2 (2.4)	0	
**Personal pension**	39 (46.4)	0	< 0.001
**Spouse pension**	2 (2.4)	17 (21.1)	< 0.001
**Still working**	11 (13.1)	0	0.001
**Monthly income in MAD (Euros)**			0.02
**- < 1500 (136)**	30 (36.6)	42 (56.8)	
**-1500–3000 (136–273)**	22 (26.8)	20 (27)	
**-3000–5000 (273–455)**	11 (13.4)	7 (9.5)	
**-5000–8000 (455–728)**	10 (12.2)	1 (1.4)	
**- > 8000 (> 728)**	9 (11)	4 (5.4)	
**Medical cover**			< 0.001
**-Ramed**	38 (45.2)	62 (78.5)	
**-CNOPS**	9 (10.7)	3 (3.8)	
**-CNSS**	11 (13.1)	3 (3.8)	
**-Military**	22 (26.2)	7 (8.9)	
**-Private insurance**	2 (2.4)	1 (1.3)	
**-None**	2 (2.4)	3 (3.8)	
**Smoking**			< 0.001
**-No**	27 (32.5)	67 (83.3)	
**-Weaned**	52 (62.7)	0	
**-Active**	4 (4.8)	0	
**-Passive**	0	13 (16.3)	
**Alcohol use**			< 0.001
**-No**	67 (79.8)	80 (100)	
**-Weaned**	17 (20.2)	0	
**-Active**	0	0	
**Osteoporosis**	1 (1.2)	23 (29.1)	< 0.001
**Food**			< 0.001
**-Cooking + errands**	9 (11)	11 (13.8)	
**-Cooking**	2 (2.4)	18 (2.5)	
**-Cooking made by another person**	71 (86.6)	51 (63.8)	
**Sexual activity**	24 (28.9)	6 (7.5)	< 0.001
**Financial difficulties**	61.66 ± 39.36	76.30 ± 38.08	0.01

There were significant differences according to the G8 status. Patients with abnormal G8 did less standing prayer, wet ablutions and fasting. They also had lower scores of physical, emotional and social functions and higher scores of financial difficulties There was no significant association between sociodemographic factors and G8 status (Table 7).

**Table 7:** Comparison in daily habits, religious practice and QOL scores according to G8 status

	G8 > 14	G8 < or = 14	p
**Illiterate**	20%	80%	0.84
**Living**			0.24
*Urban	17%	83%	
*Rural	24.6%	75.4%	
**Living alone**			0.76
*yes	0%	100%	
*No (with spouse or children)	79.4%	20.6%	
**Prayer**	93.9%	84.7%	0.25
**Standing prayer**	63.6%	19.8%	< 0.001
**Sitting prayer**	36.4%	68.7%	0.001
**Lying prayer**	3%	8.4%	0.29
**Ablutions**	97%	92.7%	0.69
**Wet ablutions**	84.8%	56.9%	0.003
**Dry ablutions**	18.2%	40.7%	0.017
**Ramadan fasting**	57.6%	21.5%	< 0.001
**Optional fasting**	45.5%	15.3%	< 0.001
**Sexual activity**	39.4%	13.1%	< 0.001
**ADL (Physical function)**	68.48 ± 30.51	42.13 ± 30.72	< 0.001
**Emotional function**	77.27 ± 29.84	57.94 ± 31.16	0.002
**Social function**	96.46 ± 15.45	75.51 ± 32.62	< 0.001
**Financial difficulties**	52.52 ± 44.11	73.33 ± 36.98	0.01

The patients who had RAMED as medical coverage had higher scores of financial difficulties, in comparison with the other patients who had AMO or military medical coverage regimen. Emotional function score was also more altered among RAMED patients (Table 8). The difference in the other QoL scores was not statistically significant.

**Table 8:** Significant differences of QoL scores according to Medical coverage Regimen

	RAMED (***N*** = 100)	AMO and Military Medical Coverage (***N*** = 55)	P
**Emotional function**	58.33 [33.33; 83.33]	75.00 [58,33; 100.00]	0.003
**Financial difficulties**	100.00 [100; 100]	33.33 [33.33; 75.00]	< 0.001

## Discussion

This is the first study especially designed to explore the lifestyle and general clinical data of elderly Moroccan cancer patients. Our results suggest that they are well supported socially but vulnerable economically. This vulnerability is more pervasive among women. The large majority of patients needed the CGA, which was not performed. Religious practice is an important daily activity for our elderly patients and represents a good indicator of fitness.

Aging results from the interaction of multiple processes. Studies suggest that cellular senescence and biological mechanisms of aging happen at approximately 75 years of age [11]. The age at retirement, tasks and social activities are important factors that influence aging. Therefore, the threshold of “old age” varies between different populations [11]. The cut-off generally used in clinical studies conducted in western populations is approximately 70 years old. In countries with a young population, studies mostly use the age of 65 years old as a cut-off. In our study, we used 65 years old as a threshold to include patients.

Our study reflects the sociodemographic characteristics of the elderly population in Morocco. In accordance with the published results of national surveys [5], this population subgroup is being urbanized and has strong family relationships, explaining the low percentage of divorce and the high proportion living with their children. This can also be explained by the low income, which does not allow bearing the expenses of another house. Therefore, children live with their parents even after marriage, and they help each other physically and financially. Cultural and religious issues also play a part as in Arabic culture and in Islam children should take care of their parents until death. Sometimes, these strong relationships may be extreme and become overly protective as observed in the 15 to 40% of families who asked physicians to hide the diagnosis from the patient [14, 19].

The proportion of patients with a high aaCCI score is low compared with other populations [20, 21] . This could be explained by the fact that there are many underdiagnosed comorbidities. Diabetes and hypertension are the most-screened comorbidities by patients and physicians. This may in part justify why polypharmacy was also less common in our patients [20–23]. This can also be related to difficulties of access to care and medications.

The fall rate is comparable with that of other populations [24]. Therefore, this should be screened for in all patients, especially when they are receiving neurotoxic chemotherapy.

The large majority of our patients had an abnormal G8 score, which reflects the need for integration of a comprehensive geriatric assessment in our elderly patients. This ratio was higher compared with other studies [16, 21, 25, 26].

To evaluate the level of dependence, we separately explored some activities of daily living (toileting, clothing, bath, movement around the house) and three instrumental activities (displacement outside the house, cooking and errands). We did not use the well-known international tools as they have not been validated on the Moroccan population yet. There are some validated geriatric tools in available classical Arabic, but they cannot be understood by our patients, who need the Moroccan dialect version of Arabic. Moreover, the QLQ-C30 scores provided us with an insight on physical and cognitive conditions. According to this study, it seems that elderly Moroccans with cancer are independent in basic daily activities. However, because in Moroccan culture cooking is considered a female task, male patients are dependent on others for cooking. Compared with other populations, elderly Moroccans have better social scores but lower scores in other geriatric domains. The “good” social scores could be explained by family and neighbor visits and the fact that the majority live with their children. Due to the lack of regular income, financial difficulties are a very important issue as shown here and in other studies [27, 28].

To our knowledge, this is the first time religious practice has been included in the assessment of the elderly in a Muslim population. We showed that religious activity was a good indicator of their level of fitness, and we conclude that it should be part of the Moroccan version of the CGA by integrating as ADL or instrumental activity of daily living (IADL).

The difference between men and women in socio-demographic characteristics is significant because of multiple factors. Unlike women, Moroccan men often remarry after the death of their spouses, which also explains why men were more sexually active. Formerly, schooling when possible was male-exclusive, as was outside work. Thus, it is extremely rare to find an old Moroccan woman with her own pension. All of that explains why there were more female patients with RAMED and low income.

The study highlights the specificity of elderly Moroccan cancer patients as an Arab and Muslim population. We realized that a big part of the Occidental version of the CGA is not suitable for our patients. For example, the majority of our patients are unable to do calculations and drawing tests. Religious practices are considered as an important activity and should be included in the Moroccan CGA.

This study represents the first designed exclusively to explore the characteristics of older Moroccan patients with cancer. The questionnaire was designed to collect their data, not to make a CGA. This explains the main limit of our study which is the lack of use of validated questionnaires (except from G8 and EORTC QLQ C30). In the context of designing our project to develop a Moroccan version of the CGA, ongoing studies are underway to develop local validated questionnaires and tests. Another limit is exhaustiveness. The number of included patients does not reflect the real flow of elderly patients in medical oncology departments. This is due to the insufficient number of physicians involved in the study and to their busy schedule of clinic and hospitalization activities. Finally, we recognize that there is a minor Arab and non-Muslim population for whom the evaluation of Muslim religious practice is not feasible. This issue should be more explored to combine the Arab culture and the other spiritual beliefs in an adapted CGA for this specific subgroup.

## Conclusions

This is the first multicenter prospective study designed to provide insight on the medical, sociodemographic, economic, clinical and vulnerability profile of older Moroccan cancer patients. Our patients are socially supported. However, there is a deep economic vulnerability, especially among women who are forced to take refuge in the family. These results illustrate how the characteristics of geriatric cancer patients are deeply influenced by cultural differences, which confirms the utility of the ongoing project to develop the Moroccan-adapted version of the Comprehensive Geriatric Assessment.

## Supplementary information

**Additional file 1.**

## Data Availability

The datasets used and/or analyzed during the current study are available from the corresponding author upon reasonable request.

## Abbreviations

Vs: Versus
RAMED: Régime d’Assistance MEDicale (Medical assistance Scheme)
CNOPS: Caisse Nationale des Organismes de Prévoyance Sociale (National Fund of Social Welfare Organizations)
CNSS: Caisse Nationale de Sécurité Sociale (National Social Security Fund)
Mutual health insurance FAR: Mutual health insurance of Royal Armed Forces
aaCCI: Age-adjusted Charlson Comorbidity Index
PS: Performance status
GCS: Granulocyte colony stimulating factor
ACCES: Accès aux médicaments pour les patients à revenus modestes (Access to drugs for patients in need)
ADL: Activities of daily living
IADL: Instrumental activities of daily living
