# Supplementary material for: Cancer in Moroccan elderly: the first multicenter transverse study exploring the sociodemographic characteristics, clinical profile and quality of life of elderly Moroccan cancer patients
Source: BMC Cancer. 2020 Oct 12;20:983. doi: 10.1186/s12885-020-07458-0 (PMC7552478; doi:10.1186/s12885-020-07458-0)
Supplement: Supplementary file 1 — Additional file 1. [file 12885_2020_7458_MOESM1_ESM.docx]

City : IP :

Center: University Hospital  Regional Center   Proximity Center Military hospital

Context : Day hospital for chemo session Consultation Hospitalization Emergency

**I/-General Data :**

**Sociodémographic data:**

Sex: Male   Female

Age:

Religion : Muslim Other if other, please specify:…………….

Civil status: Single  Married divorced Widower

Number of children :

Level of education : Illiterate mosque Elementary High School

University

Profession :

Personal income : Personal pension Spouse pension Children aid

Still working other

Monthly income : <1500MAD  1500-3000MAD 3000-5000MAD

5000-8000MAD >8000MAD

Living: urbain rural

Medical coverage: Ramed CNOPS CNSS Military

Health insurance None

Living : Alone with spouse with chidren Nursing home

Accompanying to the hospital: None 1 person >1 person

Request by the family to hide the diagnosis from the patient: non oui

**Toxic habits :**

Smoking: Never Former Active

Alcohol use : Never Former Active

Use of medicinal plants: No Yes

**II/-Daily habits :**

Toilet: Independent Partially dependent Totally dependent

Clothing: Independent Partially dependent Totally dependent

Bath: Independant Partially dependent Totally dependent

Walking indoor: Independent Partially dependent Totally dependent

Walking outdoor: Independent Partially dependent Totally dependent

Food: Cooking + errands Cooking Cooking made by another person

Number of meals per day : 3 meals + snacks 3 meals Less than 3 meals

Prayer : no standing Sitting  Lying

Ablutions : no Wet Dry

Ramadan fasting: no   yes

Optional fasting: no more than 4 days per month Less than 4 days per month

Sexual activity : no yes

**III/-Clinical data**:

**Comorbidities:**

Hypertension : no yes

Diabetes : no yes

Dyslipidemia : no yes

Cardiopathy : no yes

Osteoporosis : no yes

Arthrosis : no yes

Renal injury : non yes

Age adjusted Charlson Comorbidity Index (http://tools.farmacologiaclinica.info/index.php?sid=37146):

- Age range :

Δ <50 Δ50-59 Δ60-69 Δ70-79 Δ80-89 Δ90-99

- AIDS.
- Metastatic solid cancer.
- Liver disease moderate or severe.
- Lymphoma.
- Leukemia.
- Non metastatic solid cancer.
- Diabetes Mellitus with end organ dammage.
- Moderate or severe chronic kidney disease.
- Hemiplegia
- Uncomplicated Diabetes.
- Mild liver disease.
- Peptic ulcere disease.
- Connective tissue disease.
- COPD.
- Dementia.
- Cerebrovascular accident.
- Peripheral vascular disease.
- Myocardial infarction.
- Congestive heart failure.

One year survival according to aaCCI:

Fracture in the previous year: no yes

Falls : No yes Number

Complication : minor major

Number of medications per day : 0 < 3 ≥3

**Cancer data:**

Localization : H&N Breast col endomètre ovaire poumon esophagus stomach colon rectum kidney bladder prostate STS bone sarcoma skin pancréas Liver biliary tract CUP Other

Stage : Localized Locally advanced Metastatic at the outset Locoregional relapse Metastatic relapse

Status :1^st^ consultation work-up in progress Under treatment Follow-up

Treatment : Chemotherapy Radiotherapy concomittant chemoradiotherapy BSC

The patient has declined oncological treatment : no yes

Treatment  : Standard adjusted doses Systematic use of G-CSF less toxic and less efficient treatmet ( for example : monotherapy instead of polychemotherapy, carboplatin instead of cisplatin)

Strategy : curative palliative

**Pain and clinical exam :**

PS :

Douleur (EVS) :

Poids :

Taille :

**Screening for frailty : G8**

(<https://www.siog.org/files/public/g8_english_0.pdf>)


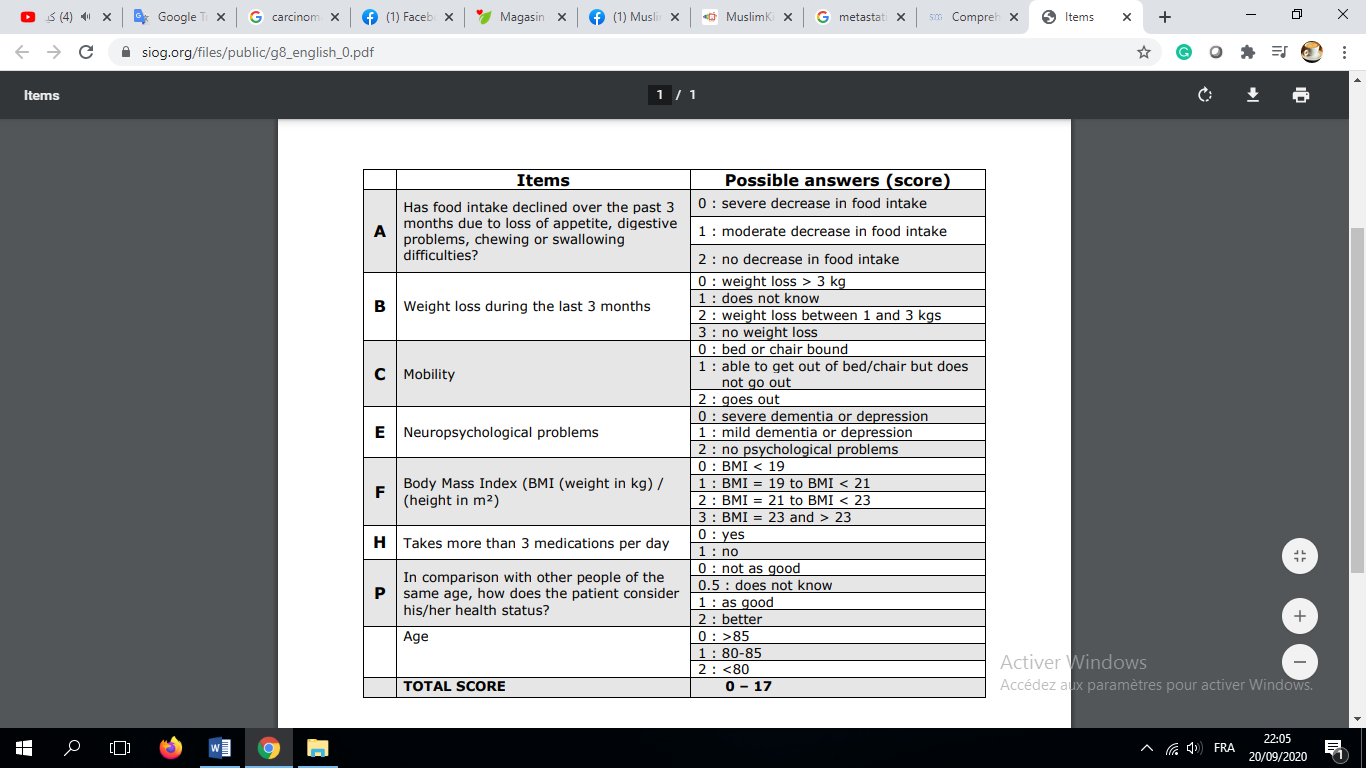


**Score :**

**IV/-Quality of life : EORTC QLQ-C30.**

We have used the Moroccan validated version.

This is a link to the original English version :

https://qol.eortc.org/questionnaire/eortc-qlq-c30/
